# An aerated axenic hydroponic system for the application of root treatments: exogenous pyruvate as a practical case

**DOI:** 10.1186/s13007-018-0310-y

**Published:** 2018-06-13

**Authors:** Miriam Gil-Monreal, Manuel Fernandez-Escalada, Mercedes Royuela, Ana Zabalza

**Affiliations:** Departamento Ciencias del Medio Natural, Universidad Pública de Navarra, Campus Arrosadía, 31006 Pamplona, Spain

**Keywords:** Amino acid biosynthesis-inhibiting herbicides, Pea roots, Ethanol fermentation, Axenic hydroponic system, Root treatments

## Abstract

**Background:**

Hydroponic systems are a convenient platform for plant cultivation when treatments are applied to the roots because they provide precise control of the composition of the growth medium, ensuring the availability of different compounds. A problem arises when axenic conditions are needed but the treatment of choice (exogenous organic acids or sugars) promote the growth of unwanted microorganisms. Moreover, axenic conditions are usually applied in liquid and semi-liquid growing systems, where oxygen availability can be compromised, if no aeration is provided.

**Results:**

The driver for the development of this hydroponic system was the application of the organic acid pyruvate to the roots of plants grown under aerated axenic conditions. No contamination was detected in the nutrient solution, even after the addition of pyruvate. The system was validated in pea plants treated with either pyruvate or herbicides inhibiting amino acid biosynthesis. The effects on ethanol fermentation were compared by analysing the enzymatic activity, protein content and transcriptional levels in plants treated with either pyruvate or herbicides.

**Conclusions:**

The developed system enables the study of the exogenous application of organic acids in the nutrient solution under axenic conditions and without oxygen limitation. This system allows the study of the effect of any type of treatments applied to roots under aerated axenic hydroponic systems at physiological and molecular levels. The role of pyruvate in the induction of fermentation by herbicides cannot be simply explained by an increase in substrate availability.

**Electronic supplementary material:**

The online version of this article (10.1186/s13007-018-0310-y) contains supplementary material, which is available to authorized users.

## Background

Hydroponic systems for plant cultivation are often used in research because they facilitate the rapid growth and homogeneity of plants and allow high reproducibility of experiments [1, 2]. Hydroponic systems are of particular interest when treatments are applied to the roots because they provide precise control of the composition of the growth medium, facilitating the management of different compounds in the medium [3–6]. The problem with these systems is that they are not efficient for the application of treatments such as exogenous applications of organic acid or sugar that boost the growth of unwanted microorganisms, treatments for which axenic conditions are needed to prevent contamination [7, 8]. Traditionally, when the applied treatments consist of the addition of organic acids or sugars, research has been performed under aseptic conditions in protoplasts [9], cell cultures [10, 11], or in seedlings grown in liquid media where the whole plant is in contact with the growth solution [12]. Growing tissues or cells in liquid or semi-liquid mediums can compromise the oxygen available to the cells if no external aeration is provided.

An axenic hydroponic system that permits an examination of the whole plant and allows the application of treatments to the roots is desirable to study the specific effects on plants of treatments applied to the soil and absorbed through the roots, such as herbicides, growth promoting bacteria, fertilizers, phytohormones, additives etc. Multiple axenic hydroponic systems have been described. A hydroponic system without aeration for aseptic conditions was developed for *Arabidopsis* [3], and more recently, an improved axenic system for the rapid production of roots has been described [8]. The latter consists of a system with a unique air source to which all the individual boxes containing the plants are connected; thus, although it is a good system for the rapid growth of roots, the system does not easily handle the application of treatments in the nutrient solution. Moreover, expanded clay balls were used to anchor the seedlings [8], which can interfere with applied treatments by adsorption of organic molecules due to the surface of clay particles.

A major driver for improving axenic hydroponic systems has been the ability to apply exogenous pyruvate to roots. Pyruvate is the substrate of pyruvate decarboxylase (PDC), the first enzyme of the ethanol fermentation pathway. Induction of aerobic fermentation has been described to be a common physiological effect of two types of amino acid biosynthesis-inhibiting herbicides (ABIHs), which are inhibitors of branched-chain or aromatic amino acid biosynthesis pathways [6, 13–16]. Due to their efficacy, ABIHs are among the most extensively used herbicides [17, 18]. Although the specific site of action of both types of herbicides was identified, the precise physiological processes that lead them to the death of the plant remain under research. Pyruvate has been proposed to regulate the fermentative response in plants treated with ABIHs [6, 13–15]. Ethanol fermentation was induced in the roots of pea plants after exogenous pyruvate application [19]. However, the fermentation induction could not simply be explained by increased pyruvate availability but instead was related to a drop in the internal oxygen concentration [15]. All these previous findings were obtained using pea roots grown in aerated hydroponic systems but not under aseptic conditions. As pyruvate promotes bacterial growth in the media, we were interested in an axenic hydroponic system that allows the exogenous pyruvate application under aseptic conditions and without oxygen limitation (aerated), to test whether the regulation of ethanol fermentation by pyruvate was also detected without potential contamination of surrounding microorganisms and to test whether the increase in pyruvate had an herbicidal effect (or enhancement of it) in plants.

Here, we describe an easily implementable hydroponic culturing system that is suitable for the application of different treatments to the root system under axenic conditions, with external individual aeration ensuring oxygen availability. In this system, all the boxes can be handled individually, facilitating the application of different treatments to individual plants. We also present a practical example in which the presented system could be put into practice, which consists of the exogenous application of pyruvate for the evaluation of its possible role as a signal in the regulation of the ethanol fermentation in plants after herbicide treatment.

## Methods

### Axenic hydroponic system

**Fig. 1:**
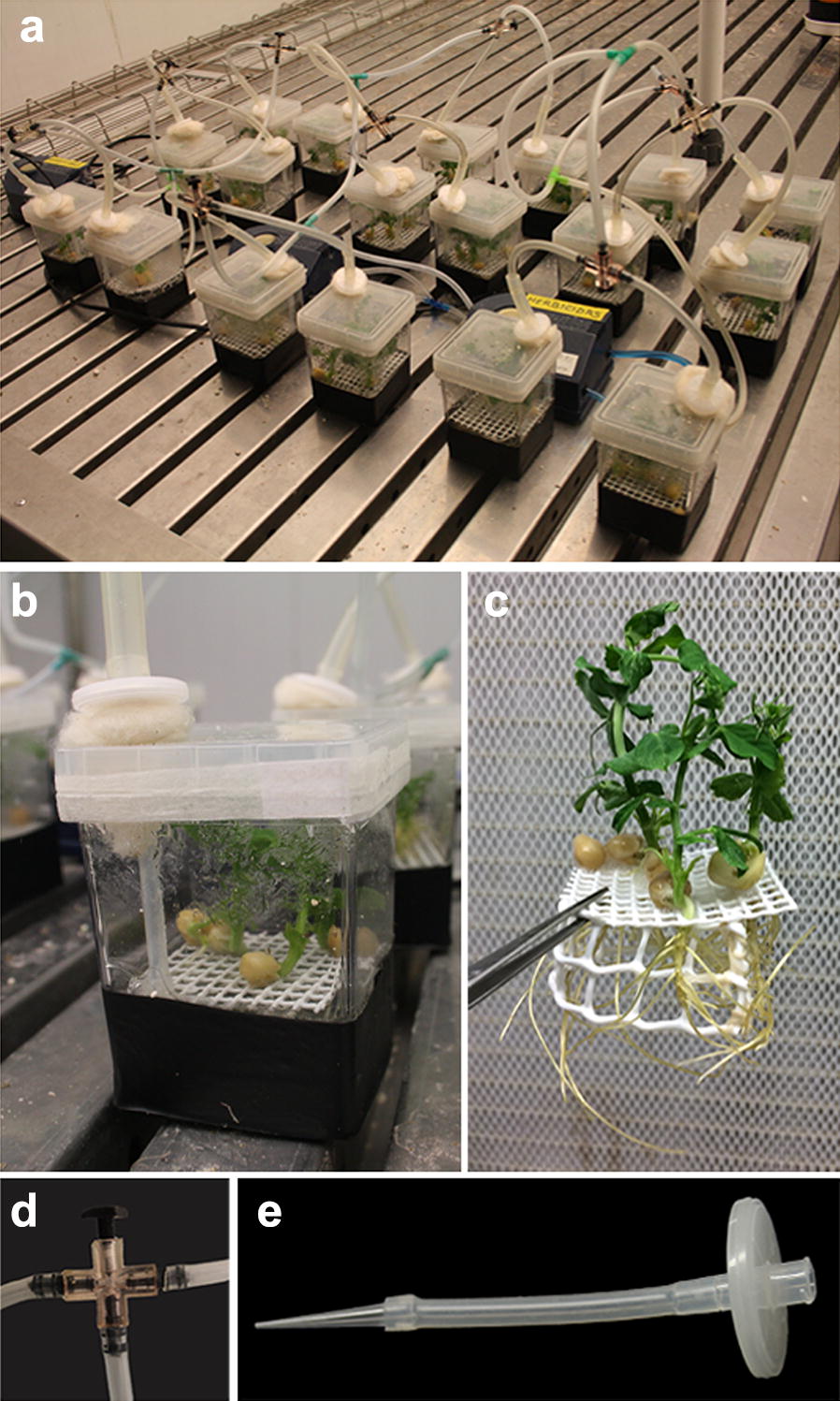
Overview of pea plants growing in the presented axenic hydroponic system. **a** Plants in the boxes were placed in a growth chamber with a 120–150 μmol m^−2^ s^−1^ light, 23/18 °C day/night temperatures and a 12/12 h day/night cycle photoperiod. The liquid medium was continuously aerated (with a flux of approximately 125 ml min^−1^) by fitting six individual boxes to a 3.7 W Elite 802^®^ air pump (Rolf C. Hagen Inc., Montreal, Canada). **b** Detailed view of the plants growing axenically in magenta boxes filled with continuously aerated nutrient solution. The bottoms of the boxes were covered with black plastic to reduce the exposure of the roots to light. An autoclavable silicone rubber tube was introduced through a hole drilled in the lid of the vessel to bubble air into the nutrient solution. Hydrophobic cotton held the tube in the hole and avoided overpressure provoked by the incoming air. The lid was sealed with Leukopor^®^ non-woven tape. **c** Autoclavable plastic mesh that holds the seeds to avoid submergence and maintains them in contact with the nutrient solution. **d** Air splitter control valve that regulates the pressure of the air bubbling the nutrient solution. **e** Autoclavable silicone rubber tube connected to a 200 µL pipette tip at the end of the tube to bubble the nutrient solution. A 0.22 µm Millex^®^ vent filter is connected to the top of the tube for the sterilisation of the incoming air

Magenta G7 boxes (Sigma-Aldrich Co., St. Louis, MO, USA) were used to grow the plants in an axenic hydroponic system (Fig. 1a). An autoclavable plastic mesh was introduced into the boxes to hold the seeds and maintain their contact with the water or nutrient solution while avoiding submergence (Fig. 1c). Water or nutrient solution was added to the boxes up to the level of the mesh containing the seeds. The bottoms of the boxes were covered with black plastic to reduce the exposure of the roots to light (Fig. 1b). To prevent the roots from developing hypoxia, the nutrient solution was continuously aerated. A hole was drilled in the lid of the vessel for insertion of an autoclavable silicone rubber tube (Sigma-Aldrich Co., St. Louis, MO, USA) connected to a 200 µL pipette tip at the end of the tube to bubble air into the nutrient solution (Fig. 1e). To hold the tube in the hole of the lid and avoid over-pressurisation by the incoming air, a piece of hydrophobic cotton was utilised (Fig. 1b). To sterilise the incoming air, a 0.22 µm Millex^®^ vent filter (Merck Millipore Ltd., Billerica, MA, USA) was connected to the tube upstream of where the air entered the box (Fig. 1e). Another tube was connected to the outer part of the vent filter, to which the air pump was connected later. Each individual box, including the vent filter and the mesh, was covered with MilliWrap autoclavable film (Merck Millipore Ltd., Billerica, MA, USA) and autoclaved. Once cooled in a laminar flow hood, the seeds were sown, and the hydroponic apparatus was then fitted with a 3.7 W Elite 802^®^ air pump (Rolf C. Hagen Inc., Montreal, Canada) and placed in the growing chamber. To control the pressure of the air, an air splitter control valve (Rolf C. Hagen Inc., Montreal, Canada) was utilised (Fig. 1d). Six boxes were connected to each pump and they were continuously aerated with an individual flux of approximately 125 ml min^−1^. Leukopor^®^ non-woven tape (BSN medical GmbH, Hamburg, Germany) was used to seal the lids.

### Plant material and treatment application

*Pisum sativum* L. cv. snap sugar boy peas were surface sterilised according to [20]. To ensure axenic conditions, all reactives were first sterilised in an autoclave or by filtering with a 0.22 µm hydrophilic Minisart^®^ syringe filter (Sartorius Stedim Biotech GmbH, Goettingen, Germany), and all of the manipulations were performed under a horizontal laminar flow cabinet. The seeds were sown in the plastic mesh (Fig. 1c) and placed in the axenic hydroponic apparatus filled with sterile water.

Plants in the boxes were placed in a growth chamber with the following growing conditions: 120–150 μmol m^−2^ s^−1^ light, 23/18 °C day/night temperatures and a 12/12 h day/night cycle photoperiod. Four days later, the water was replaced with a sterile nutrient solution described in [21] and supplemented with 10 mM KNO_3_ [14].

Treatments were applied when the plants were 6 days old. At this time point, the nutrient solution was renewed. For the herbicide treatments (ABIHs), imazamox was used as an inhibitor of the biosynthesis of branched-chain amino acids and glyphosate was used as an inhibitor of the biosynthesis of aromatic amino acids. Imazamox and glyphosate were added to the nutrient solution using commercial formulations. Final concentrations were determined based on previous studies and were 5 mg of the active ingredient L^−1^ (16.33 µM) for imazamox (Pulsar^®^40, BASF Española SA, Barcelona, Spain) [6] or 53 mg of the active ingredient L^−1^ (232.27 µM) for glyphosate (Glyfos^®^, Bayer CropScience, S.L., Paterna, Valencia, Spain) [16]. Pyruvate was supplied to the nutrient solution at a final concentration of 10 mM and replenished every 2 days (Na-pyruvate, Sigma-Aldrich Co., St. Louis, MO, USA) [19]. Some plants were exposed to low-oxygen conditions, and for that purpose, aeration was removed and the nutrient solution was bubbled with filtered N_2_ gas for 5 min every 12 h until the end of the experiment (3 days). Another set of plants was not treated and was used as the control for the treated plants. To avoid contamination, both herbicides and the pyruvate were filtered (with a 0.22 µm filter) before being added to the nutrient solution. All the manipulations were performed under a horizontal laminar flow cabinet, and all material was sterilised before being used.

For the analytical measurements, intact root samples were taken at day 3 after the application of the treatments. Plant material was immediately frozen in liquid nitrogen and stored at − 80 °C for further analysis. Later, the frozen samples were ground under liquid nitrogen using a Retsch mixer mill (MM200, Retsch^®^, Haan, Germany), and the amount of tissue needed for each analysis was separated and stored at − 80 °C.

### Presence of microorganisms test

To test for the presence of microorganisms in the nutrient solution, samples of the growth media from all treatments were taken and placed onto commercial potato dextrose agar (PDA) (pH 5.6) (Laboratorios Conda S.A., Torrejón de Ardoz, Madrid, Spain) or onto Lennox L broth media (LB) (pH 7.5) (Sigma-Aldrich Co., St. Louis, MO, USA). PDA plates were incubated at 25 or 35 °C, and LB plates were incubated at 30 or 37 °C for 3 days. As positive controls for each treatment, the nutrient solution from a box aerated with non-filtered air was used. For each box, two replicates were used.

### In vitro activities of pyruvate decarboxylase (PDC) and alcohol dehydrogenase (ADH)

The in vitro activities of PDC and ADH were assayed in desalted root extract. PDC and ADH were assessed spectrophotometrically by evaluating the NADH consumption and formation at 340 nm respectively, as described in a previous study [13]. Eight biological replicates were used for enzyme activity assays.

### PDC and ADH protein immunoblot assay

The total protein was isolated from roots as described in a previous study [14]. Protein blots were performed according to standard techniques, as previously described [19]. Goat anti-rabbit IgG conjugated to alkaline phosphatase (Sigma-Aldrich Co., St. Louis, MO, USA) was used as the secondary antibody at a dilution of 1:20,000. Cross-reacting protein bands were visualised using the Amplified Alkaline Phosphatase Goat Anti-Rabbit Immun-Blot^®^ Assay Kit (Bio-Rad Inc., Hercules, CA, USA) according to the manufacturer’s instructions. The intensities of the bands were quantified using a GS-800 densitometer (Bio-Rad Inc., Hercules, CA, USA). For immunoblot assays four biological replicates were used.

### Quantitative real-time polymerase chain reaction (qPCR)

The total RNA was extracted from approximately 0.1 g fresh weight of ground frozen roots using a phenol–chloroform extraction protocol [22]. The total RNA was subjected to a DNase treatment using the RQ1-DNase kit (Promega Biotech Ibérica, SL., Alcobendas, Spain). Five hundred nanograms of RNA was reverse transcribed into cDNA using the iScript™ cDNA Synthesis Kit (Bio-Rad Laboratories Inc., Hercules, CA, USA) following the manufacturer’s instructions. The qPCR amplification was carried out with the ABI Prism 7300 sequence detection system (Applied Biosystems, Life Technologies, Darmstadt, Germany] as described in a previous study [23]. β-*TUBULIN3* (X54846) was used as the reference gene [24]. The primer pairs used in the qPCR amplification are presented in Table 1. The relative quantifications of the expression of each individual gene were performed using the 2^−ΔΔCT^ method [25]. Transcript level analyses were performed using four biological replicates.

**Table 1:** The list of primers used in the qPCRs

	Forward	Reverse
*PDC1* (Z66543)	ggactataccggctttgtgagtgc	accttcgcagtccagcatttcc
*PDC2* (Z66544)	atgcacaagcggtacccgag	tttctggccacatcgcagca
*ADH1* (X06281)	atggcaactacaagccccgc	agctccagctcccccttcat
*β*-*TUBULIN3* (X54846)	ttgggcgaaaggacactatactg	caacatcgaggaccgagtca

### Statistical analysis

The data obtained from this study were analysed using the IBM SPSS Statistics (v.22) software package. Data are presented as the mean ± SE, which was calculated using samples from different individual plants as replicates. One-way ANOVA was used to determine the significance of the differences. The HSD Tukey and Dunnett T3 post hoc statistical tests were applied to determine the homogeneity and non-homogeneity of variances cases, respectively. In all cases, statistical analyses were conducted at a significance level of 5% (*p* < 0.05).

## Results

### Confirmation of the axenic conditions

**Fig. 2:**
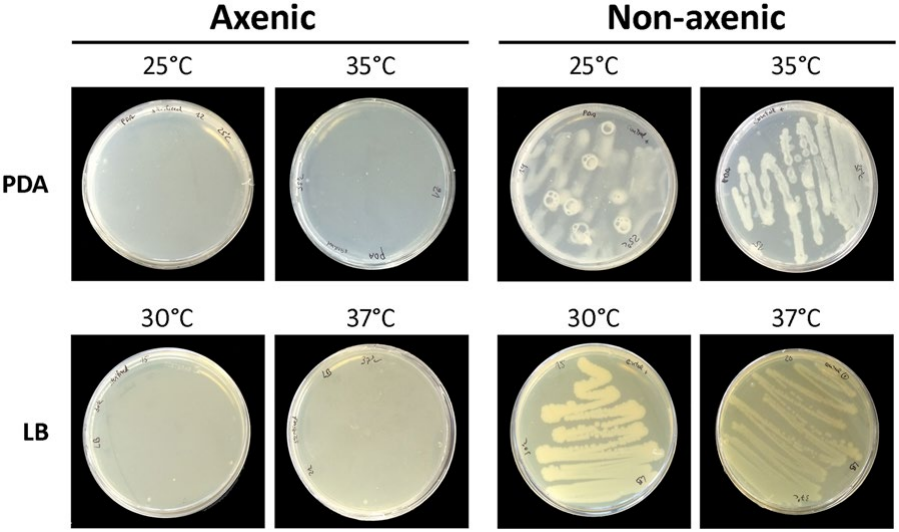
Test for the presence of microorganisms in the nutrient solution. Samples of the nutrient solution were cultured on PDA and LB plates for 3 days at different temperatures to test for the presence of microorganisms. A positive control consisting of a nutrient solution bubbled with non-sterilised air was utilised. Figure shows the cultivation of the nutrient solution containing pyruvate under axenic and non-axenic conditions as the most representative example. *LB* Lennox L broth media, *PDA* potato dextrose agar

The organic acid pyruvate is a central metabolite that can be used as fuel in many pathways and can also lead to undesirable growth of microorganisms when the nutrient solution is contaminated. In this study, pea plants were grown in an aerated axenic liquid medium to prevent contamination. To confirm that no microorganisms were present in the nutrient solution, the presence of fungi, bacteria and yeast was tested by the cultivation of nutrient solution samples on PDA and LB agar plates at different temperatures (Fig. 2). The results confirmed that no microorganisms were present in the nutrient solution of any of the boxes of this experiment (either untreated or treated), even after the addition of pyruvate to the media. By contrast, when non-sterile conditions were created by removing the air filter from the incoming air supply, microorganism contamination could be detected in the nutrient solution. As the most representative example, the cultivation of the nutrient solution containing pyruvate under axenic and non-axenic conditions is shown (Fig. 2).

### Effects on the ethanol fermentation pathway

Ethanol fermentation was measured in the roots of pea plants growing in the axenic hydroponic system. Specifically, the activities of PDC and ADH, the protein content and mRNA transcription levels were monitored in the plants. Pyruvate was supplied to the nutrient solution, and the response to this treatment was compared with the response of plants to imazamox or glyphosate application to the nutrient solution. To check that the presented system did not compromise the oxygen levels in the nutrient solution, low-oxygen stress was added as an extra treatment and was used as a positive control for fermentation activation.

**Fig. 3:**
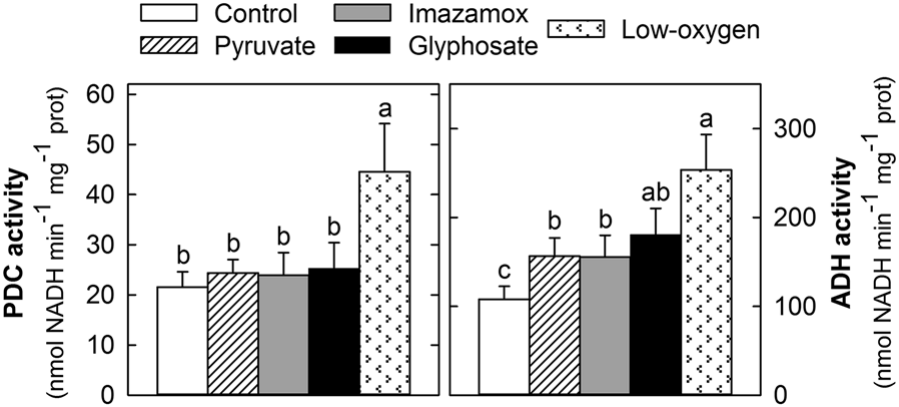
Enzymatic activities of PDC and ADH in pea roots. In vitro pyruvate decarboxylase (PDC) and alcohol dehydrogenase (ADH) enzymatic activities in desalted root extracts of plants grown in sterile conditions and harvested after 3 days of application of the treatments: untreated (control) roots; roots treated with pyruvate, imazamox or glyphosate; and roots grown under low-oxygen conditions. Mean ± SE (n = 8). Different letters indicate significant differences between treatments (ANOVA, HSD Tukey/T3 Dunnet; *p* < 0.05)

The in vitro activities of PDC and ADH were measured in plants that were untreated; treated with pyruvate, imazamox or glyphosate; or exposed to low-oxygen conditions for 3 days (Fig. 3). The low-oxygen treatment provoked an increase of the in vitro activities of both PDC and ADH in the roots (Fig. 3), confirming that the control plants were not under anaerobic conditions. In contrast, pyruvate or herbicide application only induced the activity of ADH, and no increase in the activity of PDC was detected after the applications of the three different treatments (Fig. 3).

**Fig. 4:**
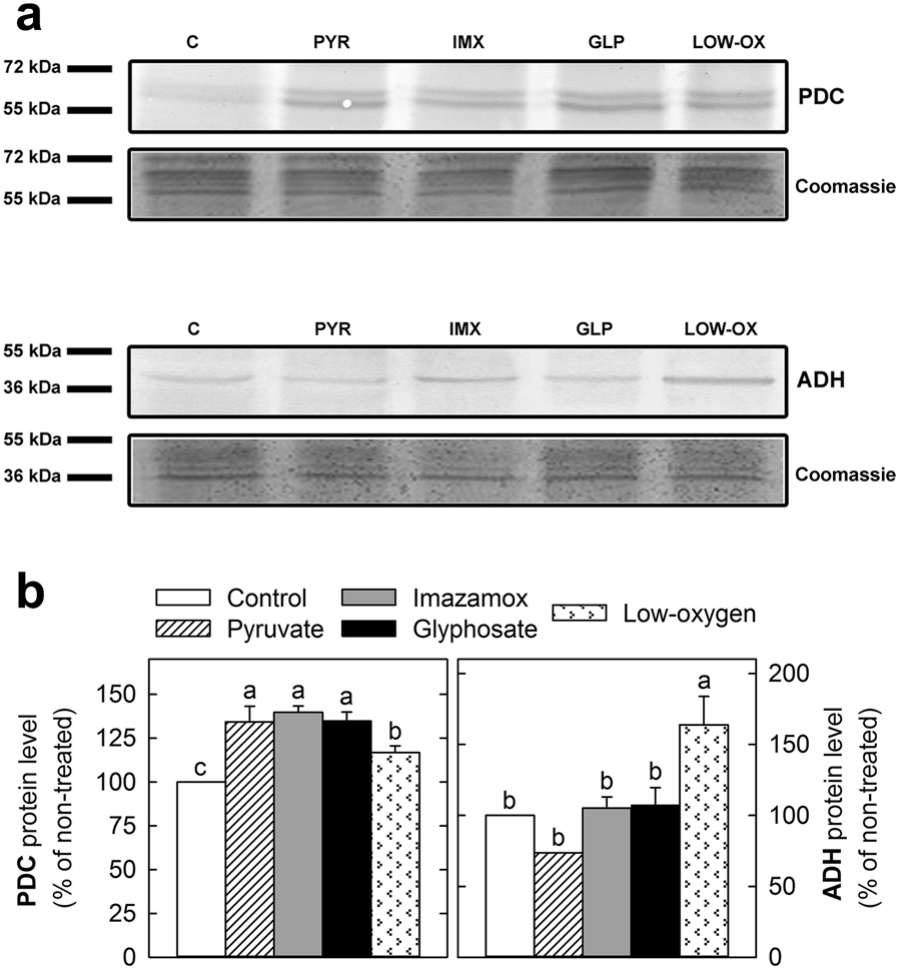
Immunoblot detection of PDC and ADH in pea roots. Plants were grown in sterile conditions and were harvested after 3 days of application of the treatments: untreated (control) roots; roots treated with pyruvate, imazamox or glyphosate; and roots grown under low-oxygen conditions. Each lane contains 30 µg of protein. **a** Protein blots for PDC and ADH. For each treatment, one representative sample is shown. The Coomassie-stained protein gel on the bottom of each blot shows the total amounts of input proteins. **b** Analyses of band intensity of blots are presented as the relative ratio to the control. The control is arbitrarily presented as 100%. Mean ± SE (n = 4). Different letters indicate significant differences between treatments (ANOVA, HSD Tukey/T3 Dunnet; *p* < 0.05). *ADH* alcohol dehydrogenase, *C* control, *GLP* glyphosate, *IMX* imazamox, *LOW-OX* low-oxygen conditions, *PYR* pyruvate, *PDC* pyruvate decarboxylase

Immunoblot analyses were carried out on the roots of pea plants treated for 3 days to evaluate whether the different treatments affected the protein content of PDC and ADH (Fig. 4). The protein content of both PDC and ADH increased in the plants exposed to low-oxygen conditions compared to the levels in the control plants, demonstrating that no oxygen limitations were exhibited in the control plants. In contrast, the protein levels of PDC increased as a consequence of pyruvate, imazamox or glyphosate application, while the ADH protein content was not modified as a consequence of either ABIH or pyruvate application.

**Fig. 5:**
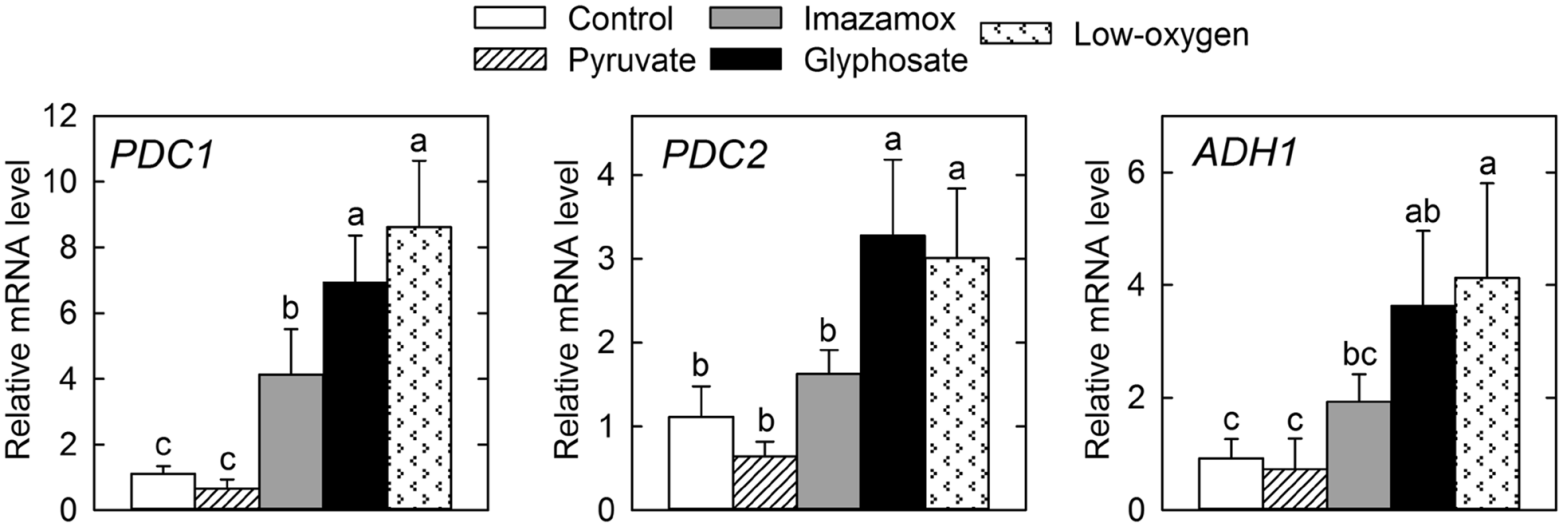
Relative transcription levels of the genes *PDC1*, *PDC2* and *ADH1* in pea roots. Plants were grown in sterile conditions, and they were harvested after 3 days of application of the treatments: untreated (control) roots; roots treated with pyruvate, imazamox or glyphosate; and roots grown under low-oxygen conditions. Mean + SE (n = 4). Different letters indicate significant differences between treatments (ANOVA, HSD Tukey/T3 Dunnet; *p* < 0.05). *ADH* alcohol dehydrogenase, *PDC* pyruvate decarboxylase

ADH has been widely studied in higher plants [26] and in peas, in which two unlinked *ADH* loci (*ADH1* and *ADH2*) express three dimeric isozymes [27]. Two PDC subunits are coded in peas by *PDC1* and *PDC2* genes [28]. To analyse whether the different treatments affected the expression of the genes involved in ethanol fermentation, the transcription levels of *PDC1*, *PDC2* and *ADH1* were measured by qPCR in the roots of pea plants (Fig. 5). The low-oxygen stress provoked an increase in the expression of the three evaluated genes, indicated by increased mRNA transcription levels of *PDC1*, *PDC2* and *ADH1* in plants exposed to anaerobic conditions. In contrast, the relative transcription levels of *PDC1* increased after application of both herbicides, while the mRNA level of *PDC2* and *ADH1* was only upregulated by glyphosate application (Fig. 5). Exogenous pyruvate application did not modify the mRNA transcript levels of the three monitored genes (Fig. 5).

## Discussion

### Validation of the method

This work presents an improved axenic hydroponic system for the growth of small plants or seedlings. Since this method is a closed system, it is important to ensure that sufficient aeration is provided to the roots and that there is no oxygen limitation for the plants. To confirm that the growing system did not compromise the oxygen availability for the plants, the activity, protein levels and mRNA transcription levels of the enzymes involved in ethanol fermentation were measured in plants exposed to hypoxia and were compared with the levels in the control plants. As expected, when aeration was removed from the boxes, the roots showed an increase in the activity of both PDC and ADH (Fig. 3), increased protein levels (Fig. 4) and an accumulation of the genes coding for these enzymes (Fig. 5). These results validate the growing method since they confirm that plants grown in this system only show signs of ethanol fermentation if the aeration is removed.

The presented system focuses on keeping axenic conditions even with aeration and the application of treatments, such as organic acids and sugars, that could promote the growth of unwanted microorganisms. To ensure that axenic conditions were maintained after the application of the different treatments, the presence of fungi, bacteria and yeast was tested by culturing nutrient solution samples on PDA and LB plates, two generic growth media used for the cultivation of microorganisms. No microorganisms were detected in the nutrient solution, even after the addition of pyruvate to the media. By contrast, when the incoming air was not sterilised, the nutrient solution was contaminated (Fig. 2). These results validate the presented hydroponic system for the application of treatments that require axenic conditions.

In addition, the typical visual symptoms provoked by these herbicides could be detected in the treated pea plants. Imazamox-treated plants exhibited root thickening, growth arrest and darkening, while glyphosate application provoked upper leaf chlorosis (Additional File 1: Fig. 1), as has been previously reported in plants treated with these types of herbicides [29, 30]. Thus, it was validated that the presented system is also suitable for the specific study of effects on plants (independently of the presence of microorganisms). This study is very interesting in the case of treatments that are usually applied to the soils and absorbed through the roots, as it is the case with herbicide application or other chemistries (fertilizers, additives, soil amendants, etc.).

### Pyruvate as a signal regulating the ethanol fermentation in plants treated with herbicides

The present method is of great interest for the exogenous application of compounds that are easily metabolized by microorganisms, and can therefore boost their unwanted growth. In this work, a practical case is presented as an example, which consists of the exogenous application of pyruvate to evaluate whether the induction of fermentation after herbicide treatment in plants can be provoked by a higher availability of pyruvate. The originality of the study lies in the exogenous application of pyruvate under aerated sterile conditions.

An increase in the activities of PDC and ADH and their protein contents has been described in the roots of pea plants treated with pyruvate and after acetohydroxyacid synthase inhibition in the branched-chain amino acid pathway by imazamox or 5-enolpyruvylshikimate-3-phosphate synthase (EPSPS) inhibition in the aromatic amino acid pathway by glyphosate [6, 16, 19]. We thus investigated whether ABIH application also provoked an activation of ethanol fermentation in pea plants grown in the presented axenic system. The detected increases in the PDC protein amount (Fig. 4) and ADH activity (Fig. 3) confirmed that ethanol fermentation was induced after the application of ABIHs in the plants grown in the presented system. In *Arabidopsis* roots, the induction was observed to be regulated at a transcriptional level since the mRNA transcription levels of the *PDC1* and *ADH1* genes increased in plants treated with ABIHs [31]. This study suggests transcriptional regulation as a general step in the induction of ethanol fermentation by ABIHs, as an induction of the transcription of both *PDC* and *ADH* genes was observed in the pea plants (Fig. 5).

The induction of fermentation after the inhibition of the synthesis of branched-chain amino acids can be associated with an increase in the pyruvate availability since this metabolite is a common substrate for both the enzyme specifically inhibited by the herbicides (acetohydroxyacid synthase) and PDC (the first enzyme in the ethanol fermentation pathway). Although induction of fermentation after glyphosate application cannot be so easily explained by an increase in the pyruvate availability, since the enzyme specifically inhibited by the herbicide (EPSPS) is not a direct pyruvate-consuming enzyme, it can be proposed that the deregulation of the biosynthetic pathway caused by glyphosate causes a massive carbon influx that increases pyruvate availability as a cross-physiological effect. Indeed, an increase in the pyruvate levels has been reported in plants after acetohydroxyacid synthase or EPSPS inhibition [6, 23].

Whether pyruvate accumulation is the only cause or only part of a cascade of signals inducing ethanol fermentation after herbicide treatment remains to be elucidated. To check whether this metabolite is a key regulator in the induction of fermentation in plants after ABIH treatment, whether exogenously supplied pyruvate regulates the fermentation in the same way as it is regulated after ABIH application was investigated. To this end, the pattern of ethanol fermentation after exogenous pyruvate supplied to the plants grown in the presented axenic hydroponic system was studied. Moreover, the effect of exogenous pyruvate was transcriptionally evaluated. Although pyruvate effects on protein content and enzymatic activity have been previously described, to the authors’ knowledge, this study describes for the first time the effect of pyruvate on the transcription of the enzymes involved in ethanol fermentation pathway.

The exogenous application of pyruvate produced effects resembling those of the herbicides with the important difference that no changes in the transcription levels of *PDC1*, *PDC2* or *ADH1* were detected (Fig. 5). ADH activity was induced after pyruvate addition, while no simultaneous increase of PDC activity was detected (Fig. 3). Interestingly, the induction of the amount of PDC enzymes after pyruvate treatment (Fig. 4) was detected, despite the absence of a higher transcription level. This effect cannot be explained only by higher substrate availability; therefore, other post-transcriptional regulation mechanisms must be involved. Collectively, the results confirmed that pyruvate participates in the regulation of ethanol fermentation. Nevertheless, as the pattern of induction of ethanol fermentation is different after pyruvate addition and after ABIH application (with transcriptional regulation in one case and without it in the other), the ethanol induction after ABIHs cannot be explained only by higher pyruvate availability (provoked by pyruvate not being consumed by the enzymes inhibited by ABIHs). Indeed, fermentation can also be regarded as a general physiological response after a stress situation (such as ABIH application), as has been reported for other abiotic stresses, such as low temperature and osmotic stress [32, 33]. These two different explanations are, however, not mutually exclusive and may even act in concert.

## Conclusions

The presented method provides an improved aerated axenic hydroponic system that facilitates the application of different treatments to the roots of plants. This system prevents plants from developing a lack of oxygen, as external sterilised aeration is provided to ensure oxygen availability. The system is of particular interest for the application of treatments such as the addition of organic acids or sugars that stimulate the growth of unwanted microorganisms, since aseptic conditions are maintained. Our system uses individual axenic boxes, which avoids the contamination of the whole experiment and facilitates the manipulation of the boxes individually. In contrast to other hydroponic systems, treatments can be applied to roots through the nutrient solution, avoiding the use of any type of substrate for anchoring the seedlings, which can interfere with the availability of the treatment. Abundant leaf or root material can be easily obtained under axenic conditions. A practical case has been used to validate the system, and interesting results are presented, supporting the conclusion that the effects on fermentation observed after ABIH treatment were due to the treatments and not to the growing method. Pyruvate has been tested under axenic conditions, showing that the role of pyruvate in the induction of fermentation after ABIH treatment is complex and cannot be simply explained by a mimicking effect or a higher substrate availability.

## Additional file

**Additional file 1.** Visual effects of herbicides. Plants treated with glyphosate presented chlorosis in the upper leaves, while imazamox provoked root thickening, growth arrest and root darkening in treated plants. Pictures were taken 17 days after treatment with 5 mg active ingredient L−1 (16.33 μM) of imazamox (Pulsar®40, BASF Española SA, Barcelona, Spain) or 53 mg active ingredient L−1 (232.27 μM) of glyphosate (Glyfos®, Bayer CropScience, S.L, Paterna, Valencia, Spain).

## Abbreviations

ABIHs: amino acid biosynthesis-inhibiting herbicides
ADH: alcohol dehydrogenase
EPSPS: 5-enolpyruvylshikimate-3-phosphate synthase
LB: lennox L broth
PDA: potato dextrose agar
PDC: pyruvate decarboxylase
qPCR: quantitative real-time polymerase chain reaction
