# Supplementary material for: An aerated axenic hydroponic system for the application of root treatments: exogenous pyruvate as a practical case
Source: Plant Methods. 2018 Jun 13;14:48. doi: 10.1186/s13007-018-0310-y (PMC5998518; doi:10.1186/s13007-018-0310-y)
Supplement: Supplementary file 1 — Additional file 1. Visual effects of herbicides. Plants treated with glyphosate presented chlorosis in the upper leaves, while imazamox provoked root thickening, growth arrest and root darkening in treated plants. Pictures were taken 17 days after treatment with 5 mg active ingredient L−1 (16.33 μM) of imazamox (Pulsar®40, BASF Española SA, Barcelona, Spain) or 53 mg active ingredient L−1 (232.27 μM) of glyphosate (Glyfos®, Bayer CropScience, S.L, Paterna, Valencia, Spain). [file 13007_2018_310_MOESM1_ESM.pdf]

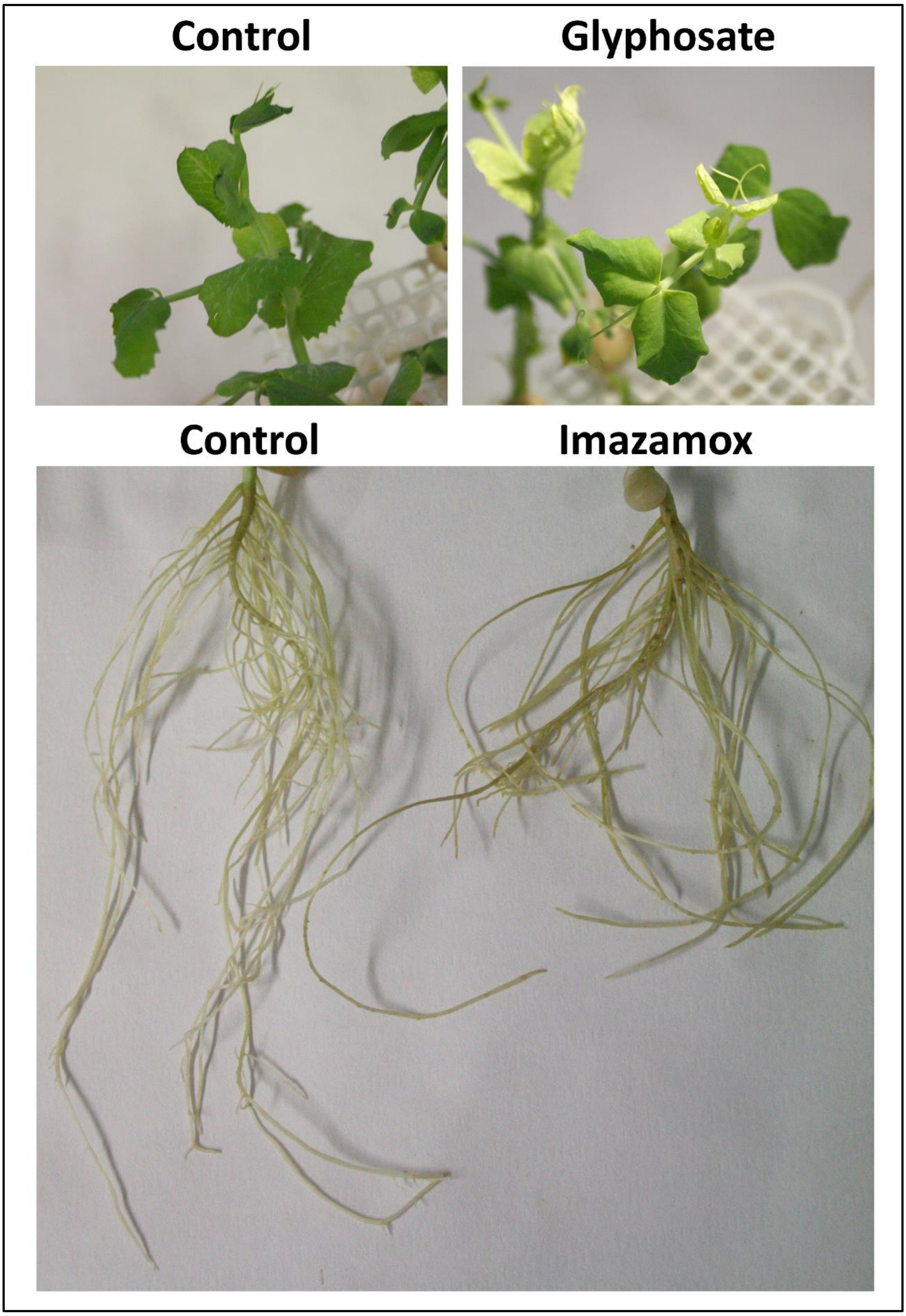

Additional File 1. Visual effects of herbicides. Plants treated with glyphosate presented chlorosis in the upper leaves, while imazamox provoked root thickening, growth arrest and root darkening in treated plants. Pictures were taken 17 days after treatment with 5 mg active ingredient L^{-1} (16.33 µM) of imazamox (Pulsar®40, BASF Española SA, Barcelona, Spain) or 53 mg active ingredient L^{-1} (232.27 µM) of glyphosate (Glyfos®, Bayer CropScience, S.L, Paterna, Valencia, Spain).
